# Cross-cultural adaptation and psychometric validation of the Arabic version of the Orofacial Awakening Symptoms Questionnaire (OFASQ)

**DOI:** 10.22514/jofph.2026.050

**Published:** 2026-07-12

**Authors:** Christelle Haddad, Ziad Azar, Anthony Mathias, Lynn Fayad, Tia Azar, Tylor Cosgrove, Mirna Fawaz, Sahar Obeid, Feten Fekih-Romdhane, Souheil Hallit

**Affiliations:** ^1^School of Medicine and Medical Sciences, Holy Spirit University of Kaslik, 466 Jounieh, Lebanon; ^2^School of Psychology, University of South Australia, 5000 Adelaide, SA, Australia; ^3^School of Nursing, Beirut Arab University, 1107 Beirut, Lebanon; ^4^Social and Education Sciences Department, School of Arts and Sciences, Lebanese American University, 1401 Jbeil, Lebanon; ^5^Department of Psychiatry “Ibn Omrane”, The Tunisian Center of Early Intervention in Psychosis, Razi Hospital, 2010 Manouba, Tunisia; ^6^Faculty of Medicine of Tunis, Tunis El Manar University, 1007 Tunis, Tunisia; ^7^Applied Science Research Center, Applied Science Private University, 11931 Amman, Jordan

**Keywords:** Orofacial pain, Orofacial Awakening Symptoms Questionnaire, Psychometric properties, Validation, Arabic

## Abstract

**Background**: Making a measure of orofacial pain available for use among Arabic-speakers might contribute to the development of prevention and therapeutic programs that consider psychosocial and behavioral determinants of the Lebanese population. This study aimed to translate, culturally adapt, and validate the Arabic version of the Orofacial Awakening Symptoms Questionnaire (OFASQ) among Lebanese adults. **Methods**: A cross-sectional web-based survey was conducted during August–September 2025 and included 427 participants (mean age = 27.5 ± 10.6 years; 56% women). The OFASQ was translated to Arabic using the forward–backward translation protocol. **Results**: Confirmatory Factor Analysis (CFA) supported a single-factor structure with good fit indices (χ^2^/*df* = 2.67, Comparative Fit Index (CFI) = 0.970, Tucker-Lewis Index (TLI) = 0.941, Root Mean Square Error of Approximation (RMSEA) = 0.144 (90% Confidence Interval (CI): 0.086–0.207), and Standardized Root Mean Square Residual (SRMR) = 0.033). Internal consistency was good (Cronbach’s α = 0.85). Measurement invariance across gender was established, with females scoring higher than males on the OFASQ. For criterion validity, higher OFASQ scores were significantly associated with worse sleep quality, more insomnia severity, and higher migraine. **Conclusions**: The Arabic OFASQ displayed reliability, validity, and cultural appropriateness for evaluating orofacial awakening indicators of bruxism and temporomandibular disorders in Arabic-speaking populations, validating its use in both clinical and research settings.

## 1. Introduction

Orofacial pain represents pain that originates from intraoral structures or the face, head, or neck and includes common conditions such as temporomandibular disorders and neurovascular headaches, including migraine [1]. A large percentage of the general population suffers from chronic orofacial pain (OFP), which often involves interacting mechanisms of peripheral nociception and central sensitization. A diagnostic system, such as the International Classification of Orofacial Pain (ICOP), separates primary from secondary causes and informs clinical decision-making [1, 2]. A multidisciplinary evaluation remains essential due to the complex musculoskeletal, neuropathic, and centralized mechanisms underlying symptom manifestations [2]. Sleep problems, mainly characterized by poor sleep quality and insomnia, commonly co-occur with pain, psychological distress, and disability in OFP [3, 4]. Neck disability is also common among people with OFP, reflecting shared musculoskeletal and psychosocial contributors to pain and functional limitation [5]. Migraine is the most common pain condition associated with OFP and is associated with insomnia and disrupted sleep, which contribute to increased headache frequency, as well as heightened disability [6]. Given the interlinked pathways between OFP, sleep problems, and migraine, there is a need for an appropriate scale that measures orofacial symptoms promptly after waking up to enhance early identification, clinical decision-making, and targeted management.

Several pain assessment instruments have been designed and utilized for the assessment of OFP, but each of them has important limitations in terms of validity and comprehensiveness. Unidimensional measures, such as the Visual Analogue Scale (VAS), Numerical Rating Scale (NRS), and Verbal Rating Scale (VRS), are often applied to quantify pain intensity and have demonstrated high sensitivity and reliability in OFP patients [7]. However, these scales are limited to pain intensity orientation and do not encompass multidimensional qualities, such as pain quality, emotional distress, functional limitation, and temporal variation—factors that are particularly relevant in long-standing OFP disorders [8]. To overcome such limitations, multidimensional instruments like the McGill Pain Questionnaire (MPQ) and the Graded Chronic Pain Scale (GCPS) have been employed to assess the qualitative and psychosocial components of chronic pain [9]. While broader in scope than unidimensional scales, these instruments were not specifically created for OFP populations and have untested psychometric characteristics in this population—such as construct validity, responsiveness, and minimal clinically important difference—which remain insufficiently established. The GCPS, for instance, is useful for grading disability, but has only fair test–retest reliability and limited cross-cultural validation in OFP populations [9]. Condition-specific measurements, such as the Jaw Functional Limitation Scale (JFLS), were then created to quantify mandibular function in Temporomandibular Disorder/Orofacial Pain (TMD/OFP) populations [10, 11]. Although the JFLS offers a focused approach to jaw function, its measurement error properties—such as smallest detectable change and responsiveness—as well as its structural validity remain insufficiently established [10]. In addition, specialized instruments exist for vulnerable groups, including the Orofacial Pain Scale for Non-Verbal Individuals (OPS-NVI) [12], but these tools show low sensitivity in patients with mild cognitive impairment, precluding their use as independent screening instruments in clinical practice [12]. Despite the presence of many pain measurement instruments, those currently used for OFP remain limited by unidimensionality, non-specificity, absence of psychometric validation, and restricted generalizability to heterogeneous groups of patients.

The Orofacial Awakening Symptoms Questionnaire (OFASQ) offers several distinct advantages over to other existing pain scales for orofacial conditions [8, 13]. The scale has demonstrated good psychometric properties, with a Cronbach’s α of 0.82 reflecting coherent measurement of the underlying construct of orofacial awakening symptoms. Construct validity was supported by a unidimensional factor structure, consistent with the theoretical framework underlying the scale [13]. Convergent validity was demonstrated by significant correlations with morning orofacial discomfort, perceived sleep quality, and self-reported sleep bruxism indicators [14, 15]. Test–retest reliability, assessed over a short interval, showed good temporal stability; the Intraclass Correlation Coefficients (ICCs) were within acceptable limits for early-stage instrument development [13]. While many instruments focus on overall pain intensity or disability felt during the day, the OFASQ evaluates orofacial symptoms in the morning, thus bridging a gap in earlier tools that tend to address frequency or presence, but not morning awakening severity of symptoms [13]. This matters because wake-episode symptoms can be prognostic of the severity and prognosis of disorders such as TMD-associated pain [13]. Second, the OFASQ uses a numerical rating scale for each item, rather than dichotomous “yes/no” or categorical response format, allowing consequently for more nuanced quantification of severity of symptoms—an advantage not offered by other instruments, such as the BruxScreen or the Standardized Tool for the Assessment of Bruxism (STAB), which record presence/frequency, but not intensity [13]. Third, its brevity and usefulness in clinical practice enhance its use for daily dental or orofacial pain clinics [13]. Finally, as a symptom-awakening instrument, the OFASQ would provide greater sensitivity for the detection of masticatory system stress or nighttime parafunction earlier than more broad daytime scales [13]. Overall, the OFASQ supplements existing measures by adding a precise, quantifiable measure of morning orofacial symptom severity and, thus, may improve screening, monitoring, and research into orofacial pain and bruxism-related disorders [13].

To date, the OFASQ has been developed and validated in English, and an Italian version has also been developed using a traditional forward-backward translation process. No formal translation, cross-cultural adaptation, or psychometric validation of the OFASQ into other languages has been reported in any peer-reviewed studies [13]. Cross-cultural validation of the OFASQ in Arabic is warranted, as orofacial awakening symptoms are influenced by language, cultural expression of symptoms, and local health-seeking behaviors. Use of a questionnaire that has not been linguistically and culturally adapted may lead to misclassification and biased prevalence estimates in Arabic-speaking populations [13]. The OFASQ was specifically developed to capture the occurrence and functional impact of awakening symptoms, addressing a gap in standard bruxism evaluation tools [13, 16]. Validation and translation of OFASQ into Arabic would, therefore, (1) allow harmonization with other Arabic translations of the TMD and bruxism measures, (2) ensure reliable assessment of symptom interference and severity for research and clinical purposes, and (3) facilitate cross-cultural comparisons and pooled analyses informing regional diagnostic thresholds and treatment planning [17, 18]. Past successful Arabic translations of bruxism and oral health questionnaires demonstrate the feasibility and worth of this endeavor and warrant standard translation, cultural adaptation, and psychometric evaluation [17, 18, 19, 20]. In brief, an Arabic OFASQ would facilitate orofacial symptom detection and observation related to awakening, promote epidemiologic and clinical research in the Middle East and North Africa (MENA) region, and facilitate culturally appropriate treatment of sleep-related orofacial disorders [13, 16]. Accordingly, this study aims to translate and psychometrically validate the Arabic version of the OFASQ. Specifically, we seek to assess its factor structure using confirmatory factor analysis, evaluate internal consistency, examine measurement invariance across sex groups, and assess concurrent validity.

## 2. Methods

### 2.1 Study design

This was a cross-sectional study conducted in Lebanon between August 2025 and October 2025. Data collection was performed using an online self-administered questionnaire distributed through social media platforms. We applied a snowball sampling technique, whereby initial participants were invited to complete the questionnaire and share it with their networks, thus facilitating recruitment across different regions of Lebanon.

Eligible participants were adults (≥18 years) residing in Lebanon who were able to read and understand Arabic. Participants who were under 18 years of age, non-Lebanese, unable to read or understand Arabic, or failed to provide informed consent were excluded from this study. Participation was voluntary and anonymous. At the beginning of the questionnaire, participants provided electronic informed consent after reading an introductory statement describing the study’s purpose, confidentiality, and their right to withdraw at any time. Ethical approval was obtained from Rayak Hospital in August 2025 [21].

### 2.2 Translation and cultural adaptation of the OFASQ

The OFASQ (English) was translated and culturally adapted into Arabic following international guidelines for cross-cultural validation [17, 18, 22]: First, a forward translation was performed: one native Arabic-speaking translator with clinical knowledge of orofacial pain performed a forward translation of the original English OFASQ into Arabic [18, 22]. This step was followed by back-translation into English by another bilingual translator who was blinded to the original OFASQ. The purpose of this step was to ensure conceptual consistency and identify potential possible distortions introduced by translation. The pre-final Arabic version was administered to a small group (n ≈ 30) to ensure clarity and comprehension. Minor linguistic adjustments were made accordingly [22].

### 2.3 Minimal sample size

For factor analyses, we followed the rule of thumb of 20 participants per item of the OFASQ, aiming for a minimum sample of N ≥100, which is considered acceptable for robust confirmatory factor analyses.

### 2.4 Study measures

Data was collected by using a structured online questionnaire with two components.

The first section collected sociodemographic variables (age, gender, marital status, education, occupation, and crowding index in the household). The second section included the following validated Arabic instruments, in addition to the Arabic version of the OFASQ.

1. The Orofacial Awakening Symptoms Questionnaire (OFASQ) [13]: The measure includes five self-report items assessing orofacial awakening symptoms, (*e.g.*, facial pain or stiffness of the jaw) which are commonly linked to sleep bruxism and TMD [15]. Each item is rated on an 11-point numerical rating scale (0 = absence of symptoms to 10 = severe symptoms). The sum score is calculated as the mean of the five items, with higher scores reflecting more severe orofacial awakening symptoms.

2. The Sleep Quality Scale [23]: This scale assesses subjective sleep quality during the past seven days. Lower scores indicate poorer sleep quality.

3. The Insomnia Severity Index (ISI) [24]: The ISI is a seven-item evaluation of the nature, severity, and consequences of insomnia symptoms over the last two weeks. Items are rated on a 0 (no problem) to 4 (very severe problem) scale, and the total score spans from 0 to 28. The higher the score, the more severe the insomnia. The Arabic version has been validated among Lebanese participants and showed strong internal reliability [25].

4. The Migraine Disability Assessment Scale (MIDAS) [26]: MIDAS is a five-item scale measuring headache disability in the previous three months, with special emphasis on days lost from work, school, or regular activities due to migraine [26]. Scores can range from 0 to 270 and are higher in proportion to migraine-related disability. The Arabic version has been validated among Lebanese patients and showed high internal consistency [27].

### 2.5 Statistical analysis

We conducted a confirmatory factor analysis in R software (lavaan package). The Maximum Likelihood estimator was used because the Weighted Least Squares Mean and Variance Adjusted (WLSMV) estimator is not appropriate when an item has no responses in a given category. Model fit was assessed with several indices, including Standardized Root Mean Squared Residual (SRMR), Root Mean Square Error of Approximation (RMSEA), Tucker-Lewis Index (TLI), and Comparative Fit Index (CFI). Adequate model fit was considered for SRMR values ≤0.05, RMSEA ≤0.08, and CFI and TLI ≥0.90 [28].

Furthermore, multi-group CFA was applied on the full dataset to test measurement invariance across sex [29]. We assessed measurement invariance across sex for the model using a four-step sequence—configural, metric, scalar, strict—and reported ΔCFI, ΔRMSEA, and ΔSRMR between successive steps; invariance decisions followed common criteria (ΔCFI ≤0.010, ΔRMSEA ≤0.015, and/or ΔSRMR ≤0.010) [30]. Group differences in OFASQ scores were examined with the Mann-Whitney test.

Floor and ceiling effects were evaluated by calculating the proportion of participants scoring the lowest and highest possible OFASQ total scores. Effects were considered present if more than 15% of respondents obtained the minimum or maximum score. Internal consistency was estimated using Cronbach’s α coefficient and McDonald’s ω. Because multivariate normality was not confirmed (Mardia’s skewness = 735.79; *p* < 0.001; kurtosis = 28.19; *p* < 0.001), validity was explored through Spearman correlation coefficients between the OFASQ scale and other constructs.

## 3. Results

Participants’ details are summarized in Table 1.

**Table 1.** Sociodemographic and clinical characteristics of the participants (n = 427).

Variables		n (%) or Mean ± SD
Sex		
	Males	188 (44.0%)
	Females	239 (56.0%)
Education level		
	Primary	8 (1.9%)
	Secondary	24 (5.6%)
	University	395 (92.5%)
Marital status		
	Single	345 (80.8%)
	Married	69 (16.2%)
	Divorced	7 (1.6%)
	Widowed	6 (1.4%)
Employment		
	Full-timer	163 (38.2%)
	Part-timer	45 (10.5%)
	Unemployed	18 (4.2%)
	Student	201 (47.1%)
Age (yr)		27.52 ± 10.61
Household crowding index (person/room)		0.99 ± 0.42
OFASQ total		3.57 ± 3.85
Sleep quality		3.00 ± 0.97
Insomnia severity		11.28 ± 5.06
MIDAS total		9.57 ± 17.52

*OFASQ: Orofacial Awakening Symptoms Questionnaire; MIDAS: Migraine Disability Assessment Scale; SD: Standard Deviation.*

### 3.1 Confirmatory factor analysis

Fit indices of the one-factor model were convenient, except for the RMSEA value (Table 2). Internal reliability was satisfactory for the total score (ω = 0.85/α = 0.85).

**Table 2.** Fit indices and standardized loading factors of the Arabic version of the OFASQ items scales via confirmatory factor analysis.

Item		Loading factors
	1: Difficulties to open mouth on awakening	0.83
	2: Stiffness, fatigue or tightness in jaw muscles or in temples on awakening	0.72
	3: Pain in jaw muscles or in temple on awakening	0.81
	4: Pain in temporomandibular joint on awakening	0.88
	5: Teeth soreness on awakening	0.90
		Fit indices
χ^2^/*df*		13.36/5 = 2.67
Robust CFI		0.970
Robust TLI		0.941
Robust RMSEA (90% CI)		0.144 (0.086, 0.207)
SRMR		0.033

*SRMR: Standardized Root Mean Squared Residual; RMSEA: Root Mean Square Error of Approximation; TLI: Tucker-Lewis Index; CFI: Comparative Fit Index; CI: Confidence Interval; df: Degree of freedom.*

The OFASQ total score ranged from 0 to 17 in the present sample. A total of 134 participants (31.4%) obtained the minimum score of 0, indicating a substantial floor effect. Only one participant (0.2%) obtained the maximum score of 17, indicating no ceiling effect.

Measurement error indices were estimated using distribution-based methods. The Standard Error of Measurement (SEM) was calculated as SD × √(1 − α), yielding an SEM of 1.49. The Minimal Detectable Change at the 95% CI (MDC_95_) was computed as 1.96 × √2 × SEM = 4.13.

### 3.2 Measurement invariance

Invariance was shown across both sexes at all levels. Females (Median = 3.00, Inter-Quartile Range (IQR) = 6) had higher OFASQ scores than males (Median = 1.00, IQR = 5), U = 16,779, *Z* = −4.57, *p* < 0.001, Effect size = 0.26 (small-medium effect size) (Table 3).

**Table 3.** Measurement invariance of the OFASQ items scales across sexes (n = 188 males and n = 239 females).

Model	CFI	RMSEA	SRMR	Model Comparison	ΔCFI	ΔRMSEA	ΔSRMR
Configural	0.990	0.118	0.039				
Metric	0.993	0.085	0.041	Configural *vs.* metric	0.003	−0.033	−0.002
Scalar	0.994	0.070	0.040	Metric *vs.* scalar	0.001	−0.015	−0.001

*CFI: Comparative Fit Index; RMSEA: Root Mean Square Error of Approximation; SRMR: Standardized Root Mean Square Residual.*

### 3.3 Criterion validity

Higher OFASQ scores were significantly associated with worse sleep quality, more insomnia severity, and higher migraine (Table 4).

**Table 4.** Correlation matrix.

	1	2	3
1. OFASQ	1.00		
2. Sleep quality	−0.22***	1.00	
3. Insomnia severity	0.27***	−0.58***	1.00
4. MIDAS	0.39***	−0.17***	0.31***

***p < 0.001; numbers in the table reflect Spearman correlation coefficient (rho). OFASQ: Orofacial Awakening Symptoms Questionnaire; MIDAS: Migraine Disability Assessment Scale.

## 4. Discussion

The present study aimed to translate, culturally adapt, and psychometrically validate the Arabic version of the OFASQ in a Lebanese adult population. The findings validate the Arabic OFASQ as a psychometrically sound and culturally adapted instrument for assessing orofacial awakening symptoms, most likely related to TMD and bruxism in Arabic-speaking adults. To the best of our knowledge, this is the first validation of the OFASQ in Arabic [13].

CFA supported a unidimensional factor structure, as initially suggested in the scale development [13]. The one-factor model also demonstrated good fit indices (CFI = 0.970, TLI = 0.941, SRMR = 0.033), although the RMSEA was 0.144, indicating some degree of misfit [31]. These results suggest that the Arabic OFASQ assesses a homogeneous construct representing orofacial awakening symptoms, although small item-specific residual correlations may improve subsequent versions of the scale [31]. Moderate RMSEA values are common in short symptom instrument validation studies, particularly when all items heavily load onto a single factor [31, 32, 33]. Although the CFI, TLI, and SRMR values indicated good model fit, the RMSEA value exceeded the commonly accepted threshold (0.08). It is well established that RMSEA can become inflated in models with low degrees of freedom (*df*) [31, 32, 33]. Our model had a small degree of freedom (*df* = 17), a condition under which RMSEA tends to overestimate misfit and behave less reliably than other fit indices. Given that the other fit indices (CFI, TLI, and SRMR) met conventional criteria and factor loadings were strong, the overall model was considered acceptable [31, 32, 33].

Furthermore, internal reliability was good, with Cronbach’s α of 0.85 and McDonald’s ω of 0.85, both exceeding the minimum recommended for newly translated scales (0.70). These figures compare favorably to those of the original English validation and indicate the Arabic version maintains equivalent measurement properties across cultures [13].

Tests of measurement invariance identified configural, metric, and scalar invariance between gender groups, supporting measurement equivalence of the OFASQ across men and women [31, 34]. Women had significantly higher OFASQ scores than men, consistent with previous findings reporting higher prevalence and severity of TMD and bruxism symptoms among women [5, 35, 36]. Presumably, these findings reflect a mix of biological and psychosocial factors, such as hormonal influences and greater help-seeking and stress-reactivity [37].

Regarding concurrent validity, OFASQ scores were significantly correlated with poorer quality of sleep, greater insomnia severity, and greater migraine disability. The positive correlation with insomnia severity emphasizes the link between orofacial pain, cervical musculoskeletal tension, and sleep disturbance [38, 39, 40]. Inverse but small correlations with sleep quality suggest that, although sleep influences orofacial symptoms, this relationship may be indirectly mediated by stress and musculoskeletal mechanisms [3, 41]. Overall, these findings—observed in the Lebanese population—are consistent with the biopsychosocial model of orofacial pain, with its emphasis on interdependence among physiological, psychological, and behavioral determinants.

### 4.1 Clinical implications

The Arabic version of the OFASQ has been validated in Lebanon, providing a reliable and culturally sensitive tool for clinicians, dentists, and other sleep specialists assessing orofacial motor and sensory symptoms related to sleep and awake bruxism among Arabic speakers [17, 21]. As we see increased awareness of sleep-related movement disorders and their connection to stress, temporomandibular dysfunction, and orofacial pain [40, 41], this tool will allow for early screening and improved characterization of these symptoms in clinical and community settings.

The results of females having markedly higher scores on the OFASQ has important clinical implications. This pattern aligns with well-established epidemiological evidence showing that women report higher orofacial pain incidence, greater sensitivity to pain, and higher prevalence of TMD. These differences may arise from biological, hormonal, psychological, and sociocultural factors [37].

A key clinical consideration is whether these findings warrant gender-specific cutoff scores. At this stage, the observed differences do not justify separate thresholds, but they underscore the importance of interpreting OFASQ scores with awareness of gender-related variability. Clinicians should be aware that higher scores for females may not always reflect greater pathology, but may reflect expected baseline differences in pain reporting. Future studies are needed to validate gender-specific norms or thresholds that may improve diagnostic accuracy. Clinicians should apply the current scale uniformly pending such data but recognize that the interpretation of scores may differ between males and females. Furthermore, the validated Arabic OFASQ contributes to the standardization of assessment tools across populations to allow for cross-cultural comparisons and the development of prevention and therapeutic programs that consider the psychosocial and behavioral determinants of the Lebanese population [17].

### 4.2 Strengths and limitations

Strengths of this research include rigorous translation and adaptation procedures following established cross-cultural validation criteria [17, 22], the use of multiple validated Arabic instruments for the purpose of concurrent validity [13, 23, 24, 25, 26, 27], and the demonstration of strong internal reliability and gender invariance.

The current study also has some limitations. The cross-sectional design does not allow causal inferences about the association of orofacial awakening symptoms with psychosocial or behavioral factors, and therefore longitudinal research is suggested to determine temporal and predictive relationships. Self-report questionnaires have potential recall and response bias [41, 42], and the absence of objective clinical measures, such as electromyography or polysomnography, limits comparisons between subjective symptoms and physiological indicators. A substantial floor effect (31.4% scoring the minimum value), which reflects the low prevalence of orofacial awakening symptoms in the general population, may reduce measurement sensitivity for individuals with minimal symptoms. The pronounced floor effect is expected given the community-based sample, where many individuals do not experience orofacial awakening symptoms. However, the absence of ceiling effect suggests that the questionnaire adequately captures higher symptom levels. The use of a single forward translator deviates from standard guidelines, which recommend at least two independent forward translations [18, 22]. Because only one forward translation was produced, a formal reconciliation stage was not applicable; however, minor wording and phrasing adjustments were made internally by the research team to ensure clarity and conceptual adequacy. These internal decisions were not fully documented, which constitutes a methodological limitation. The internet-based snowball sampling probably reduced representativeness and may have excluded people with restricted internet access or lower health literacy, thus contributing to selection bias [42]. In addition, the online snowball sampling resulted in a young, student-based sample, which limits the external validity of the Arabic OFASQ and may reduce the stability of its psychometric indicators. The correlation observed with sleep quality, insomnia, and migraine [6] was significant but small in magnitude and merely provides limited support for convergent validity. In addition, sleep quality was rated using only one item and not by a valid measure of (Patient-Reported Outcomes Measurement Information System) PROMIS Sleep Disturbance, which limits the strength of evidence for construct validation. Because the study did not exclude individuals with TMD or bruxism, the sample likely included participants with and without these conditions. This would be an important distinction for the wider utility of the scale, as without the separation into clinical and non-clinical groups, it becomes more difficult to assess how well the measure generalizes across varying levels of symptom severity or diagnostic status. Temporal stability, usually gauged by test–retest reliability, forms a fundamental part of psychometric evaluation. In the present study, we did not examine the test–retest reliability and sensitivity to change of the Arabic version of the OFASQ. The study should be transparent about this limitation, since the absence of repeated measurement prevents confirmation of whether the scale yields consistent, overtime stable scores. This omission was since the online, snowball-based recruitment approach precluded, for practical reasons, any possibility of recontacting participants within a controlled interval for a second administration of the instrument. However, the absence of a test–retest assessment restricts the completeness of the validation process, and future studies should include longitudinal measurement to establish the scale’s stability. The studies that involve multimodal assessment—physiological, clinical, and psychophysical—would further enhance the evidence base for the Arabic OFASQ and support its use in broader cultural and clinical contexts. Finally, although the Arabic OFASQ showed good reliability and factorial validity in the Lebanese sample, further work is required to establish its stability across other Arab populations. Specifically, test–retest reliability and responsiveness to clinical change should be assessed through longitudinal or intervention studies.

## 5. Conclusions

This study successfully translated, culturally adapted, and psychometrically validated the Arabic version of the OFASQ [13] among Lebanese adults. The Arabic OFASQ was found to have good reliability and structural validity. The instrument exhibited correlations with sleep quality, insomnia, and migraine disability, reinforcing its concurrent validity and agreement with the biopsychosocial model of orofacial pain, especially in the younger population. The Arabic OFASQ, thus, emerges as a proper, concise, and culturally relevant instrument for the assessment of orofacial awakening bruxism and TMD symptoms in adolescents or young adults of Arabic-speaking populations. Its use within clinical and research settings can facilitate earlier diagnosis and interdisciplinary evaluation, as well as aid cross-cultural uniformity of diagnostic measures in orofacial pain and sleep-related movement disorder research.
